# Comprehensive Review and Evidence-Based Treatment Framework for Optimizing Plantar Fasciitis Diagnosis and Management

**DOI:** 10.7759/cureus.88745

**Published:** 2025-07-25

**Authors:** Tonyclinton C Nweke

**Affiliations:** 1 Podiatric Surgery, Wyckoff Heights Medical Center, New York, USA

**Keywords:** clinical practice guidelines (cpg), evidence based treatment, heel pain, initial therapies, intermediate therapies, plantar fascia, plantar fasciitis, specialised therapies, surgical therapies

## Abstract

Plantar fasciitis, a prevalent cause of heel pain, results from inflammation of the plantar fascia, often due to repetitive stress and overuse. This comprehensive review and evidence-based treatment framework for optimizing plantar fasciitis diagnosis and management aims to inform podiatric surgeons (foot and ankle surgeons), primary care physicians, orthopedic surgeons, physical therapists, sports medicine specialists, and other medical practitioners. Developed through a literature review from mostly 2020 to 2025 using PubMed and Cochrane, this framework integrates high-quality evidence in the pathophysiology and treatment of plantar fasciitis. Diagnosis relies on detailed patient history, physical examination (including palpation, windlass test, and heel squeeze test), and selective imaging (X-rays, ultrasound, or Magnetic Resonance Imaging (MRI)) to confirm plantar fasciitis and rule out differentials such as tarsal tunnel syndrome or calcaneal stress fractures. A four-phase plantar fasciitis treatment framework categorizes 30 plantar fasciitis treatments into initial therapies, intermediate therapies, specialized therapies, and last-resort surgical therapies. Initial therapies (e.g., Rest, Ice, Compression, Elevation (RICE), stretching, orthotics) focus on early symptom relief, while intermediate (e.g., photobiomodulation therapy (PBMT), extracorporeal shock wave therapy (ESWT)) and specialized therapies (e.g., platelet-rich plasma (PRP), dry needling) address persistent cases. Surgical options like endoscopic plantar fascia release are reserved for recalcitrant cases. Recommendations prioritize low-risk, high-efficacy interventions, progressing to invasive treatments only when necessary, ensuring tailored management to optimize patient outcomes and minimize complications.

## Introduction and background

Plantar fasciitis is the inflammation of a thick band of connective tissue known as the plantar fascia in the foot. The plantar fascia extends from the heel (medial tuberosity of the calcaneus) to the toes. It supports the arch of the foot and absorbs shocks during physical activity. This inflammation occurs when the plantar fascia has been overstretched. Micro tears are also noted on the plantar fascia due to overuse and repetitive stress.

It is the most common cause of heel pain worldwide. More than 10% of the world's population is affected by plantar fasciitis [1]. Plantar fasciitis affects athletes, nonathletes who stand for prolonged periods, people who use improper or unsupportive footwear, people with tight calf muscles, individuals with a flat foot (pes planus) or high arch (pes cavus), and people with high body mass index (BMI).

Plantar fasciitis presents with symptoms like heel pain, localized tenderness in the arch or heel of the foot, pain after exercise, pain upon first steps in the morning, and stabbing or sharp heel pain.

Diagnosis

To diagnose plantar fasciitis, a proper history is taken from the patient. Details such as the location of the pain, nature of the pain, onset, and duration of the pain are essential in the diagnosis.

A physical examination will be the next step in diagnosis. This entails inspecting the foot for bruising, deformities, swelling, foot type, gait abnormalities, and compensatory motion. The plantar medial calcaneal tubercle is next palpated to assess pain. Following this, the ankle joint range of motion is assessed to see if there is limited ankle dorsiflexion, which is an indication of a tight calf muscle (Achilles tendon) that can contribute to plantar fasciitis. A lateral heel squeeze test is also necessary to rule out a calcaneus stress fracture. A windlass test, also known as a jack’s test, is then performed by passively dorsiflexing the big toe of the foot, to increase the tension of the plantar fascia and elicit pain in that area. This allows the plantar fascia to be stretched and the pain reproduced in the heel further confirms plantar fasciitis. 

Imaging such as X-ray, ultrasound, or MRI is also critical in the diagnosis of plantar fasciitis. X-rays are needed to rule out any fracture of the calcaneus or bones of the foot as well as confirm heel spurs. Heel (calcaneal) spurs are common among patients with plantar fasciitis [2]. To visualize the soft tissues, an ultrasound or MRI is needed. An ultrasound will show the thickening of the plantar fascia (greater than 4 mm), micro tears, and inflammation of the plantar fascia [3]. Ultrasound also shows perifascial fluid and hypoechoic areas. An MRI is an expensive alternative to ultrasound, however, it is indicated for chronic cases. It shows fascia thickening, and increased signal intensity and can be used to rule out tumors or stress fractures. 

It is important to keep in mind that plantar fasciitis could exhibit symptoms similar to other diseases, hence the need to know the differential diagnosis, which includes: tarsal tunnel syndrome, plantar fascia rupture, calcaneus stress fracture, Achilles tendinopathy, heel pad atrophy, rheumatologic conditions, Baxter neuropathy, inflammatory arthritis, and tumors.

Methods

The development of this comprehensive review and evidence-based treatment framework for optimizing plantar fasciitis diagnosis and management involved a very rigorous approach to synthesizing evidence-based recommendations. A comprehensive literature search was performed using PubMed and Cochrane. Search terms included “plantar fasciitis” and “level of evidence”. The majority of the articles used were studies from 2020 to 2025. Over 30 high-quality studies were selected for detailed analysis based on their relevance, methodological rigor, and contribution to understanding pathophysiology, diagnostics, or treatment efficacy for plantar fasciitis. The four-phase plantar fasciitis treatment framework was developed to organize treatments of plantar fasciitis into four phases: initial therapies, intermediate therapies, specialized therapies, and last-resort surgical therapies. Evidence was graded using a Levels of Evidence framework (Table 1), prioritizing high-quality randomized controlled trials and systematic reviews. Recommendations were made by synthesizing over 30 high-quality studies, ensuring evidence-based guidance for plantar fasciitis diagnosis and management.

**Table 1:** Levels of evidence This table outlines the levels of evidence used to evaluate the quality and strength of studies used in the creation of this comprehensive review and evidence-based treatment framework for optimizing plantar fasciitis diagnosis and management. This hierarchical system categorizes research based on study design as well as methodological rigor. This is to ensure that the recommendations made in this framework are grounded in high-quality evidence. Level I includes randomized controlled trials, representing the highest level of evidence. Level II encompasses prospective studies, which provide strong evidence. Level III includes case-controlled and retrospective studies, offering valuable insights. Level IV consists of a case series. Level V represents expert opinions, providing clinical insights. This framework guided the comprehensive review and synthesis of evidence.

LEVELS	STUDY DESIGNS
Level I	Randomised controlled trials
Level II	Prospective studies
Level III	Case-controlled and retrospective studies
Level IV	Case Series
Level V	Expert Opinion

## Review

Four-phase plantar fasciitis treatment framework

Phase 1: Initial Therapies

Description: Initial therapies include non-invasive, conservative treatments designed to alleviate pain, reduce inflammation, and improve function in the early stages of plantar fasciitis which is approximately one to four weeks of symptoms. These therapies are low-risk, cost-effective, and accessible, making them ideal as first-line interventions. They focus on managing acute symptoms of plantar fasciitis and correcting the biomechanical issues without requiring the use of specialized equipment or invasive procedures.

Rationale for categorization: Plantar fasciitis treatments such as Rest, Ice, Compression, Elevation (RICE) therapy, stretching, orthotics, low-dye taping, and dry cupping are categorized as initial therapies due to their non-invasive nature, ease of implementation, and proven efficacy in addressing inflammation, mechanical stress, and tissue elasticity in the early phase of plantar fasciitis. These interventions are suitable for most patients and provide conservative management to achieve symptom resolution before considering more advanced options.

RICE therapy: RICE therapy is a first-aid therapy used to manage inflammation and acute soft tissue injuries. Since RICE is effective in managing inflammation, it can also be used in managing plantar fasciitis. Rest is employed by avoiding activities that stress the plantar fascia. Avoid walking barefoot and taking short breaks to sit, as well as wearing proper shoes that support the feet. Ice is the application of cold to the plantar fascia to reduce inflammation. The application of ice constricts the blood vessels in the affected area of the foot and numbs the area, therefore slowing down nerve conduction. An ice pack covered in thin cloth can be applied to the plantar aspect of the foot for 20 minutes before bedtime [4]. 

An experimental study by Laymon et al. examined the effect of applying cold to the plantar fascia in the morning or the night before to treat the pain and other symptoms of plantar fasciitis. Thirty patients with plantar fasciitis were examined and split into three groups of 10. One group did not receive any treatment (control), the other group used cold therapy for 20 minutes at night before bed, and the third group used cold therapy for 20 minutes in the morning right after waking up. Their plantar fascia tenderness and pain level were examined with the use of a visual analog scale (VAS), and their plantar fascia thickness was also examined with high-resolution ultrasound. Their pain tolerance was tested with an algometer, and the foot and ankle range of motion were examined. They found that applying cold at bedtime worked best for easing symptoms. They also found out that although morning cold therapy helped, it was not as effective as the evening application of cold. The bedtime cold group saw a 13% drop in plantar fascia thickness (p<0.05), a 44% reduction in pain, and an 86% increase in how much force their foot could handle without pain (p<0.05). This study concluded that applying cold therapy for 20 minutes before bedtime was more effective compared to applying cold therapy in the morning when treating the symptoms of plantar fasciitis [4].

Compression in the RICE therapy for plantar fasciitis works by the application of external pressure. An elastic bandage or compression sleeve can be wrapped around the foot to reduce inflammation. Elevation of the foot is also necessary to reduce blood flow to the area, which in turn reduces inflammation. Elevation of the foot should be above the level of the heart for 10-15 minutes, two to three times daily. The RICE therapy is necessary for acute flare-ups and is effective in reducing inflammation and pain. It is used for the first one to four weeks of the symptoms and can be used alongside other conservative therapies.

Stretching: Stretching is one of the most effective conservative therapies for plantar fasciitis [5]. It involves stretching the plantar fascia and the calf muscles (gastrocnemius and soleus muscles), which include the Achilles tendon, to elongate these structures. One of these stretching exercises is the calf (gastrocnemius and soleus) stretch. The gastrocnemius is connected by the Achilles tendon to the calcaneus which in turn influences the plantar fascia. This stretch involves keeping both feet flat on the ground and then placing the unaffected leg forward and the affected leg straight backward, then leaning forward, bending the front knee while keeping the back leg straight. The duration of this stretch is 30 seconds and can be performed two to three times per leg, twice or thrice per day.

A prospective cohort study conducted by Sugino et al. with level 2 evidence found that the plantar elasticity increased regardless of the frequency and type of plantar fascia-specific stretching. Before the stretching, the plantar fascia elasticity was between 133.8 and 144.7 kPa. However, after the stretching the plantar fascia elasticity increased between 158.9 to 215.8 kPa. Stretching led to a noticeable boost in plantar fascia elasticity (P < .01) [6].

Another study, a prospective randomized controlled trial by Kaiser et al. with therapeutic level l evidence compared physical therapy with independent home stretching for plantar fasciitis and found out that at six months, people doing home stretching experienced a drop in their pain level by 35% (highly significant, P <0.001), while those in physical therapy had a 26% pain reduction (also significant, P <0.002). Both showed strong progress however home stretching showed higher progress compared to physical therapy [7].

Orthotics: Orthotics are specialized shoe inserts that are created to support the longitudinal arch of the foot. They can be customized which is specifically tailored to the foot type and biomechanics of the patient or they can be prefabricated. Orthotics absorb shocks when a patient is standing or walking and in this case, can mitigate the effect of the pain from the plantar fasciitis [8].

A randomized clinical trial by Taseh et al., a level I study, compared the effects of three types of prefabricated insoles orthotics, namely polyethylene (PE), polyurethane (PU), and carbon fiber, for treating plantar fasciitis. Patients were assigned to use one of these insoles (14 with carbon fiber, 14 with PU, 17 with PE) and their outcomes were measured at baseline, two, six, and 12 weeks using pain and function scales (PROMIS 3a for pain intensity, PROMIS 4a for pain interference, Foot and Ankle Outcome Score (FAOS) for foot and ankle function, and VAS for pain). Both the carbon fiber and PE insoles significantly reduced pain intensity starting at six weeks (p = 0.04) and two weeks (p = 0.002), respectively, and also improved pain interference. The study concluded that prefabricated carbon fiber and PE insoles effectively reduced pain in patients with plantar fasciitis [9].

Low-dye tape: Low-dye tape is a taping technique where a rigid athletic tape is used to stabilize the arch of the foot, especially in patients with plantar fasciitis. Strips of 1-2 inches wide of this athletic tape are placed in crisscross patterns from the lateral heel, to under the arch and then to the medial forefoot. Additional strips of low-dye athletic tape can be placed to reinforce the arch of the foot with plantar fasciitis and this tape allows for mechanical change in the position of the joint [10].

A randomized controlled study by Enzin et al. compared extracorporeal shock-wave therapy (ESWT) and low-dye taping for treating plantar fasciitis. Seventy-two patients were assigned randomly to two groups. One of the groups received ESWT, while the other group received low-dye taping with sham ESWT. Each group underwent three weekly treatment sessions. Pain was measured by the VAS and their function FAOS was assessed before treatment, after treatment, and at a six-week follow-up period. Both of these groups showed a very significant reduction in pain and improvements in function at the end of treatment and follow-up (p ≤ 0.001). However, there were no significant differences in pain or function between the ESWT and low-dye taping groups at either time point (p > 0.05). The study concluded that ESWT and low-dye taping are both equally effective conservative treatments [11].

Dry cupping: Dry cupping is the use of suction cups to create negative pressure to improve blood flow to the plantar fascia [12]. This process stimulates the mechanoreceptors. As circulation is increased through this process, nutrients and oxygen are delivered to the plantar fascia. This suction cup is applied to the heel for five to 15 minutes per session, once or twice weekly for four to eight weeks.

A review by Szlosek et al. examined whether dry cupping, a treatment that uses negative pressure to boost blood flow and aid healing, is effective for managing plantar fasciitis. The study focused on whether dry cupping improves pain and function compared to therapeutic exercise or electrical stimulation. Three studies were included: two of the studies compared dry cupping to exercises and stretching, and one of the studies compared dry cupping to electrical stimulation. The findings of this study suggest moderate evidence to support the use of dry cupping to reduce pain and improve function in patients with plantar fasciitis [13].

Recommendation for Phase 1 (weeks one to eight): A comprehensive conservative treatment plan combining home-based stretching with custom or prefabricated orthotics, supplemented by RICE therapy and low-dye taping as needed, is recommended to effectively manage acute plantar fasciitis symptoms and promote early recovery. Home stretching is the cornerstone of this phase due to its superior efficacy, as demonstrated by Kaiser et al.’s level I randomized controlled trial, which reported that people who did home stretching had a 35% reduction in pain compared to those doing physical therapy who had a 26% drop in pain (P < .001) [7]. This highlights its effectiveness and accessibility for patients. Sugino et al.’s level 2 prospective cohort study further supports stretching by showing a significant increase in plantar fascia elasticity (P < .01) when stretching is done, which aids in reducing mechanical stress [6]. Bedtime RICE therapy, particularly the application of ice for 20 minutes before sleep, is recommended by Laymon et al.'s 2013 experimental study, which demonstrated a 44% pain reduction, 13% reduction in plantar fascia thickness, and 86% increase in pain-free force tolerance (P < .05) when cold therapy is applied at night time, outperforming the morning application of cold therapy for the treatment of plantar fasciitis [4]. This makes bedtime ice application a critical component for alleviating tenderness and inflammation.

Orthotics, particularly carbon fiber or polyethylene insoles, are included based on Taseh et al.’s level I randomized clinical trial, which discovered a significant reduction in pain at two and six weeks (P = .002 and P = .04, respectively), providing biomechanical support to complement stretching [9]. Low-dye taping, supported by Enzin et al.’s randomized controlled study, showed a significant pain reduction and functional improvement (P ≤ .001) and advised for additional arch stabilization if symptoms persist beyond two weeks [11]. Dry cupping, backed by Szlosek et al.’s review showing moderate evidence for pain relief, can be considered as an adjunct if needed [13]. This combined approach ensures comprehensive symptom management, leveraging the strengths of each therapy to maximize pain relief and functional restoration within eight weeks. If symptoms persist beyond this period, escalation to Phase 2 is recommended.

Phase 2: Intermediate Therapies

Description:* *This phase includes therapies that are more specialized, often involving pharmacological agents or technological interventions, but remain non-invasive except for non-steroidal anti-inflammatory drug (NSAID) injection, which is minimally invasive. These treatments are indicated for patients with persistent symptoms (beyond eight weeks) or moderate plantar fasciitis requiring targeted pain relief or tissue healing.

Rationale for categorization:* *Therapies such as NSAIDs, ESWT, photobiomodulation therapy (PBMT), low-level laser therapy (LLLT), iontophoresis, and phonophoresis are categorized as intermediate due to their requirement for clinical administration, higher cost, or use of medications/devices. These interventions target inflammation, pain, and some tissue repair through advanced mechanisms.

Non-steroidal anti-inflammatory drugs:* *NSAIDs are medications that inhibit cyclooxygenase (COX) enzymes which in turn reduce inflammation and pain. NSAID inhibits COX1 and COX2 enzymes and by doing this the synthesis of prostaglandin is reduced [14]. NSAIDs can be oral or injectable. Oral NSAIDs like Ibuprofen (400-800 mg) and naproxen (500 mg) can be taken twice daily. Injectable NSAIDs such as ketorolac (15-30 mg) can be injected near the plantar fascia. 

A study by Akram et al. compared the effectiveness of oral NSAIDs versus locally injectable steroids for treating plantar fasciitis. This study included 140 patients (aged 26-60, 72.9% male) with unilateral plantar fasciitis and moderate to severe pain, who had not received prior treatment. They were divided into groups. Group A had 70 patients who received oral diclofenac sodium (50 mg) and acetaminophen (500 mg) twice daily for four weeks, while Group B also had 70 patients who received a single injection of 40 mg methylprednisolone with 2 ml of 0.5% bupivacaine at the most tender aspect of their inflamed plantar fascia [15].

The study assessed the pain level using the VAS at baseline and after two months. Results from the study showed that both groups experienced reduced pain, however, group B which was the group that received the injected steroid showed a significant reduction (p=0.0001), while group A, which received the oral NSAIDs, did not show a significant reduction (p=0.723). The study concluded that locally injected steroids were much more effective than oral NSAIDs for reducing pain from plantar fasciitis [15].

Extracorporeal shock wave therapy:* *ESWT is a therapy that involves the use of high-energy acoustic waves to overstimulate the nerve endings and promote the formation of new blood vessels which allows fibroblasts to remodel the collagen of the damaged fascia. Shock-wave therapy stimulates cell growth and alters the expression of mRNA leading to the expression of growth factor-beta 1 (TGF-β1), collagen I, and collagen III [16]. All of these help with the healing process of the tissue. This therapy can be administered as a three- to five-session therapy one to two weeks apart.

A systematic review and meta-analysis study by Charles et al. reviewed the effectiveness of ESWT for the treatment of patellar tendinopathy (knee pain), Achilles tendinopathy (heel pain), and plantar fasciitis (foot pain). The study showed that for plantar fasciitis, ESWT significantly improved pain and function in both the short and long term. However, when ESWT was compared to other treatments such as low-level laser therapy, corticosteroid injections, or conservative treatments, the effects of ESWT on pain and function in the short term were inconclusive [17].

Photobiomodulation therapy:* *PBMT is the use of non-thermal and low-intensity light to stimulate tissue healing. This therapy also uses visible, non-ionizing, and near-infrared light to trigger cellular reactions [18]. This therapy releases light energy that is absorbed by chromophores. Through this oxidative stress is reduced, adenosine triphosphate (ATP) production is increased and pain is decreased. This device is applied to the arch of the foot and the heel for five to 15 minutes per session, twice or thrice weekly for four to eight weeks.

A systematic review and meta-analysis study by Ferlito et al. reviewed 19 randomized controlled trials with 1,089 participants to evaluate the effects of PBMT for managing pain and disability in plantar fasciitis. This study found that PBMT, either alone (Mean Difference (MD) = - 22.02 [- 35.21 to - 8.83]) or combined with exercise (MD = - 21.84 [- 26.14 to - 17.54]), significantly reduced pain in the short term. PBMT was more effective than ESWT for pain relief. Furthermore, in follow-up, PBMT in combination with exercise outperformed exercise alone for pain reduction [19].

This study also found that PBMT may improve disability in the medium and long term compared to therapeutic ultrasound, but the improvement might not be clinically significant. In addition, there’s uncertainty about PBMT’s ability to improve disability overall, and it doesn’t enhance outcomes when combined with other therapies. While PBMT effectively reduces pain, especially compared to ESWT, it doesn’t consistently outperform other treatments for pain or disability and isn’t supported as an add-on to other electrotherapeutic methods [19].

Low-level laser therapy:* *LLLT is a therapy that delivers monochromatic light to the plantar fascia for tissue repair. This therapy employs photochemistry, utilizing a specific wavelength (color) of light to trigger a signal transduction cascade by activating a light-absorbing protein [20].

LLLT stimulates the activity of the mitochondria, thereby increasing ATP production and reducing the inflammatory cytokines. Fibroblast activity is then enhanced and the remodeling of collagen takes place. This therapy is applied to the heel and plantar fascia for five to 10 minutes per session, twice or thrice per week for four to eight weeks.

A systematic review and meta-analysis of randomized controlled trials by Naterstad et al. reviewed the effectiveness of LLLT for treating lower extremity tendinopathy or plantar fasciitis. The study analyzed 10 trials comparing LLLT to placebo, five trials comparing LLLT to other interventions, and three trials using LLLT as an add-on to other interventions. LLLT significantly reduced pain and disability right after therapy was completed ((13.15 mm VAS; 95% CI 7.82 to 18.48)) and four to 12 weeks later (12.56 mm VAS (95% CI 5.69 to 19.42)). Furthermore, when using recommended LLLT doses, the pain was reduced even more compared to placebo or when added to exercise therapy, with effects lasting four to nine weeks(15.90 mm VAS (95% CI 2.3 to 29.51)) [21].

Iontophoresis:* *Iontophoresis is a therapy that uses a low-voltage electrical current to send the medication into the plantar fascia [22]. These electrodes are two in number and deliver medications like corticosteroids, acetic acid, or dexamethasone. These then target the inflammation mediators and reduce the inflammation and pain coming from the plantar fascia. A current of 1-4 mA is applied to the heel for 10-20 minutes each session, twice or thrice for two to four weeks.

A double-blinded randomized controlled trial by Pabón-Carrasco et al. compared the short-term effectiveness of iontophoresis with the use of lidocaine 0.4% and dexamethasone 0.5% versus radial ESWT for treating chronic plantar fasciitis. One hundred twenty-seven patients were studied and assigned to two groups. Group A received iontophoresis and Group B received ESWT. Outcomes, including heel pain (VAS), health status (EuroQol-5D questionnaire), and the plantar fascia thickness which was measured by ultrasound, were assessed at baseline and over five weeks [23].

The ESWT group showed significant reductions in fascia thickness and VAS pain scores (p = 0.001), achieving complete pain remission after three weeks (VAS 1.0 ± 0.9). Both the iontophoresis and the ESWT groups reached complete pain remission by six weeks. ESWT was perceived by patients as more effective by the end of treatment (p = 0.001), although both treatments were satisfactory. The study concluded by indicating that ESWT provides faster pain relief than iontophoresis; however, both treatments are effective for short-term pain reduction in plantar fasciitis [23].

Phonophoresis:* *Phonophoresis is a therapy that makes use of ultrasound waves to transdermally deliver medications into the tissue [24]. For instance, dexamethasone in the form of gel is applied to the heel then an ultrasound device with a high-frequency sound wave is used to drive the medication into the plantar fascia. This session can be done from five to 10 minutes per session, twice or thrice weekly for two to four weeks.

A randomized double-blind clinical trial study by Karakilic et al. compared the effectiveness of prolotherapy, phonophoresis, and corticosteroid injections for treating plantar fasciitis in 146 patients. The patients were randomly assigned to one of the three treatment groups. The patients were assessed at baseline, one month, and three months post-treatment using the Heel Sensitivity Index (HSI), VAS for pain, Foot Function Index (FFI), Short Form-36 (SF-36) for quality of life, and plantar fascia thickness via the use of ultrasound [25]. 

All three treatments significantly improved all measured outcomes at one and three months (p < .05). There were no significant differences in VAS or FFI scores between the three treatment groups. However, the prolotherapy group showed greater improvements in HSI (p = .021) and SF-36 general health subscale (p = .033 at one month, p < .01 at three months) compared to the phonophoresis and prolotherapy groups. The study concluded that all three treatments are safe and effective for early plantar fasciitis treatment, with prolotherapy showing longer-lasting improvements in heel sensitivity and general health, although ultrasound findings remained unchanged at three months [25].

Recommendation for Phase 2 (weeks eight to 20):* *PBMT is recommended as the primary intervention, supplemented by LLLT or ESWT if needed, to achieve significant pain reduction and promote tissue healing in patients with persistent plantar fasciitis. PBMT is prioritized due to Ferlito et al.’s systematic review and meta-analysis, which demonstrated a substantial short-term pain reduction (MD = -22.02, P < .05) compared to ESWT, and its effectiveness when combined with exercise, making it a robust choice for intermediate management [19]. LLLT is recommended as a complementary or alternative option, supported by Naterstad et al.’s systematic and meta-analysis study, which showed sustained pain reduction (13.15 mm VAS, P < .05) and improved disability four to 12 weeks post-therapy, particularly with recommended doses [21]. ESWT, backed by Charles et al.’s systematic review and meta-analysis showing significant long-term pain and function improvements, is advised if PBMT or LLLT are unavailable or insufficient, despite its inconclusive short-term superiority [17]. Iontophoresis and phonophoresis, while effective (Pabón-Carrasco et al. and Karakilic et al., P < .05), are secondary due to slower pain relief compared to ESWT [23,25]. Oral NSAIDs, as shown by Akram et al. (P = .723), are less effective than injectable steroids and should only be used as an adjunct for temporary pain relief [15]. This recommendation prioritizes PBMT for its superior evidence base, ensuring targeted pain relief and tissue repair within eight to 20 weeks. If symptoms remain unresolved, escalation to Phase 3 is warranted.

Phase 3: Specialised Therapies

Description:* *This phase includes therapies that involve injections or needle-based procedures to directly target the plantar fascia, promoting tissue regeneration or pain relief. These treatments are indicated for chronic or recalcitrant plantar fasciitis (beyond 20 weeks) that does not respond to Phases 1 and 2 interventions.

Rationale for categorization:* *Treatments such as dry needling, amniotic membrane injections, platelet-rich plasma (PRP), polydeoxyribonucleotide (PDRN), calcium phosphate, corticosteroid injections, botulinum toxin A, dextrose prolotherapy, perforating fat injections, and allogenic growth factors are categorized as specialised therapies due to their use of needles or injections, higher risk profile, and need for specialized administration. These therapies aim to repair tissue or modulate pain pathways.

Dry needling: Dry needling is a technique where filiform needles are placed into the myofascial trigger points of the plantar fascia area. This in turn disrupts the trigger points and causes muscle contractions. The pain-gating system is activated in this technique and inflammation is reduced while blood flow is improved. In addition, dry needling reduces the concentration of substance P which in this case will cause a reduction in inflammation and pain [26]. This sterile needle can measure from 0.25-0.30 mm in diameter and be placed for five to 10 minutes, once or thrice weekly for four to six weeks.

A systematic review and meta-analysis study by Llurda-Almuzara et al. reviewed whether dry needling is effective for treating plantar heel pain or plantar fasciitis. Two hundred ninety-seven publications were searched and six trials were included in this analysis. The findings showed that dry needling reduces pain in the short term by about 1.7 points on a pain scale compared to other treatments (MD -1.70 points, 95% confidence interval [CI] -2.80 to -0.60; SMD -1.28, 95% CI -2.11 to -0.44) and moderate-quality evidence that it improved pain intensity (MD -1.77 points, 95% CI -2.44 to -1.11; SMD -1.45, 95% CI -2.19 to -0.70) and related disability (SMD -1.75, 95% CI -2.22 to -1.28) in the long term when compared with the comparison group [27].

Amniotic membrane allograft injection: Amniotic membrane (AM) allograft injection involves injecting human AM fluid into the plantar fascia thereby allowing tissue regeneration. This amniotic fluid obtained from the placenta contains cytokines, extracellular matrix, and growth factors [28]. The allograft allows for the stimulation of fibroblasts and angiogenesis. Furthermore, this therapy leads to a reduction in inflammation and pain. A 1-2 mL of AM allograft is injected into the plantar fascia with the guidance of an ultrasound.

A retrospective, single-center, matched case-controlled study by Nakagawa et al. compared the effectiveness of ultrasound-guided percutaneous plantar fasciotomy (UGPF) alone versus UGPF combined with an AM allograft injection for the treatment of plantar fasciitis. In this study, 15 patients received UGPF alone, while 16 patients received UGPF plus AM injection. Results showed that the group that received UGPF plus AM showed a significant pain reduction (p = 0.02) from baseline at the short-term follow-up; however, by 52 weeks, there was no significant difference in pain or satisfaction between the groups. Conclusively, both treatments significantly reduced pain and also achieved high patient satisfaction, although combining UGPF with AM may offer a much faster pain relief early after the procedure [29].

Platelet-rich plasma therapy: PRP therapy is a therapy where a patient's platelet is injected into the plantar fascia. This platelet, which is rich in growth factors, is isolated from the patient's blood through a centrifuging process. 20-60 mL of blood is drawn from the patient and centrifuged to yield 3-5 mL of PRP, which is in turn infused into the plantar fascia of the patient. This therapy can restore the deteriorating condition occurring at the base of the plantar fascia [30].

A level 1 systematic review and meta-analysis study by Herber et al. analyzed 21 randomized controlled trials that involved 1,356 patients. PRP therapy was compared with other treatments for plantar fasciitis. The study showed that PRP significantly reduced pain in VAS pain scores compared to placebo (SMD: 3.42; CI: [2.53, 4.31]; p < 0.00001) and other therapies like ESWT (SMD: 0.86; CI: [0.30, 1.41]; p = 0.002) and corticosteroid injections (CSI) (SMD: 0.86; CI: [0.30, 1.41]; p = 0.002). It also improved foot function (measured by American Orthopaedic Foot and Ankle Society (AOFAS) scores) more than CSI (SMD: 3.31, CI: [1.35, 5.27], p = 0.0009 and placebo (SMD: 3.75; CI: [2.81, 4.70]; p < 0.00001). However, PRP showed no significant difference or advantage over ESWT, CSI, dextrose prolotherapy, or meridian trigger points in improving foot function (FFI scores) or plantar fascia thickness (PFT) [31].

PRP was shown to be much more effective than phonophoresis in improving both FFI and PFT (SMD: 3.07, 95% CI: 2.34-3.81). This study shows that while PRP is effective for reducing pain and improving some functional outcomes, its benefits vary across measures, therefore suggesting the need for standardized PRP preparation and outcome assessment in plantar fasciitis treatment [31].

Polydeoxyribonucleotide: Polydeoxyribonucleotide (PDRN) is a therapy where polydeoxyribonucleotide is injected into the plantar fascia. It is a compound that binds to adenosine receptors and stimulates angiogenesis and fibroblast activity [32]. This therapy allows the synthesis of collagen and helps with regenerating the damaged or injured plantar fascia. 5-10 mg in 1-2 mL is injected into the plantar fascia insertion for three to five sessions.

A prospective randomized clinical study with level 2 evidence by Lee et al. compared the effectiveness and safety of PDRN injection versus CSI for treating 44 patients with plantar fasciitis. Plantar fascia thickness and echogenicity were evaluated with the use of ultrasound, and complications were noted. At two and six weeks, CSI reduced pain more than PDRN (p=0.010 and p=0.016), but by six months, both treatments showed similar pain relief (p=0.523). The study concluded that PDRN injections are a safe and effective alternative to CSI for plantar fasciitis, with comparable results at six months [32].

Calcium phosphate: Calcium phosphate, which is delivered through an injection into the plantar fascia area, is an effective therapy for plantar fasciitis. Calcium phosphate will act as a scaffold to support the repair of tissues [33]. It stimulates the activity of fibroblasts, osteoblasts, and the deposition of collagen into the area. 1-2 mL of calcium phosphate cement is injected into the plantar fascia.

A level 3 retrospective comparative study by Matthew et al. compared two treatments for chronic, treatment-resistant infra calcaneal heel pain with calcaneal bone marrow edema (BME) in 64 patients treated between 2014 and 2019. The first group had 33 patients who received plantar fasciotomy alone, while the second group had 31 patients who received fasciotomy with a calcaneal subchondral injection of calcium phosphate [33].

Both groups showed significant improvements in four of five FAOS subscales after surgery. However, the group that received calcium phosphate injections had significantly better scores in activities of daily living (mean difference +10.2) and foot-specific quality of life (mean difference +21.9) at the final follow-up compared to the group with only fasciotomy. The study concluded that combining calcium phosphate injection with plantar fasciotomy is safer and much more effective than receiving fasciotomy alone for recalcitrant heel pain with BME [33].

Corticosteroid and anesthetic injections: The injection of corticosteroid and anesthetic into the plantar fascia is another way used to manage plantar fasciitis. Corticosteroid inhibits inflammatory pathways while anesthesia blocks the nerve signals. It is very common to merge both corticosteroid and anesthesia for a much better result. However, there have been concerns that excessive use of corticosteroids could lead to potential rupture of the plantar fascia [34]. A physician can inject a mixture of 0.5-1 mL of corticosteroid with 1-2 mL of anesthesia like lidocaine.

A systematic review and meta-analysis level 2 clinical evidence study by Seth et al. reviewed the effectiveness of CSI compared to PRP and ESWT for treating plantar fasciitis. It analyzed 18 studies involving 1,180 patients, sourced from databases like PubMed and Medline, following Preferred Reporting Items for Systematic Reviews and Meta-Analyses (PRISMA) guidelines, up to April 2021. Pain (VAS) and foot function (AOFAS score) were assessed [35].

At three and six months, PRP therapy resulted in lower pain scores compared to CSI with lignocaine/lidocaine (mean difference of 0.62 and 1.49, respectively). At six months, ESWT also showed better pain reduction than CSI (mean difference of 0.8). In addition, PRP therapy improved AOFAS scores more than CSI at six months (mean difference of -11.53). The study concluded that PRP and ESWT are more effective than CSI for reducing pain at three and six months, and PRP is more effective for improving function at six months. However, larger, high-quality studies are needed to solidify these findings [35].

Botulinum toxin A:* *Botulinum toxin A, also known as Botox, is a neurotoxin made by Clostridium botulinum that can be injected into the plantar fascia [36]. It reduces muscle tension. Botulinum toxin A blocks the release of substance P which is an 11-amino acid neuropeptide involved in physiological processes, like inflammation, and pain perception and transmission. Botulinum toxin A also prevents the release of acetylcholine at neuromuscular junctions, thereby causing the temporary relaxation or paralysis of muscles which in turn reduces tension in the plantar fascia.

A level I therapeutic randomized controlled double-blind study by Elizondo-Rodríguez et al. compared three treatments for plantar fasciitis: botulinum toxin A, corticosteroid, and two intralesional anesthetic injections. All patients improved in pain and function compared to their assessments before the study. It was also noted that the thickness of the plantar fascia decreased by the end of the study. Ankle dorsiflexion also improved across all the treatment groups. However, there were no significant differences between the treatment groups in any of these outcomes. Pain relief and functional improvements were sustained over the six-month follow-up, regardless of the treatment used [37].

Dextrose prolotherapy:* *Dextrose prolotherapy is a therapy that involves the injection of dextrose solution into the plantar fascia. Dextrose is a sugar solution that in this case stimulates growth factors, fibroblasts, and immune cells to repair injured and damaged plantar fascia [38]. 1-2 mL of dextrose solution is injected into the plantar fascia using a 25-gauge needle, in three to five sessions for two to four weeks is recommended.

A randomized double-blind clinical trial study with level 2 evidence by Raissi et al. compared ultrasound-guided injections of dextrose (20%) versus corticosteroid (40 mg methylprednisolone) for treating chronic plantar fasciitis in 44 patients, with 40 patients completing the trial. Both treatments significantly reduced pain and improved function at 2 and 12 weeks. At 2 weeks, the corticosteroid group had lower daytime and morning pain scores, better sports-related function, and reduced plantar fascia thickness compared to the dextrose group, though daily activity function was similar. By 12 weeks, outcomes were similar between groups. The study suggests that both treatments are effective for chronic plantar fasciitis, with corticosteroids showing better early results but similar outcomes to dextrose at 12 weeks [39].

Perforating fat injections: Perforating fat injections are the injection of fat into the heel or plantar fascia to add cushion to improve pain level and function [40]. The goal of this is shock absorption thereby reducing mechanical stress. 1-3 mL of fat is injected into the heel pad of the plantar fascia.

A level 2 randomized cross-over clinical trial study by Gusenoff et al. evaluated perforating fat injections as a treatment for chronic first group which was the intervention group had nine women who received fat injections and were followed for 12 months, while the second group had five patients, who were injected and were observed for six months. An average of 2.6 ml of fat was injected per foot at one or two sites. Outcomes included plantar fascia thickness (measured by ultrasound), pain, function (assessed via validated patient-reported measures), and complications [40].

The results of the study revealed that in group one the plantar fascia thickness decreased significantly at six and 12 months. The pain also improved at both time points, and activities of daily living and sports activities increased compared to baseline. However in group two, the plantar fascia thickness reduced six months post-injection, and sports activity improved, but pain levels remained unchanged. The study concluded that perforating fat injections significantly reduce pain, improve function, and decrease the plantar fascia thickness in chronic plantar fasciitis [40].

Allogenic growth factors: The allogenic growth factor (GF) can be injected into the plantar fascia to stimulate tissue repair and promote collagen synthesis, fibroblast activity, and angiogenesis. Some of these growth factors include VEGF, TGF-β, and PDGF. 1-2 mL of allogenic GF is injected into the plantar fascia for one to two sessions for four to six weeks.

A prospective randomized controlled case series with level I evidence by Kandil et al. evaluated the effectiveness and safety of injecting allogeneic GFs for plantar fasciitis in 150 patients. These patients were split randomly into two groups, with one of the groups receiving GF injections and the other group receiving saline treatment as their control. Pain (VAS) and foot function (Foot Function Index-Revised short form (FFI-Rs)) were utilized in measuring before injection and at one, three, six, and 12 months after [41].

At baseline, both groups had similar VAS and FFI-R scores. However, by three months, the GF group showed an 87% reduction in pain compared to a 55% pain reduction in the control group, and a 62% improvement in function compared to 40% in the control group (both significant, P < .001). Furthermore, the GF group had a higher satisfaction rate (92% vs. 78.2%). However, five patients in the GF group reported post-injection pain. The study provides strong evidence that GF injections are effective and safe for plantar fasciitis, but further research is needed to assess potential adverse effects, microbiological safety, and immune responses [41].

Recommendation for Phase 3 (weeks 20 to 52):* *PRP injections are recommended as the primary treatment, followed by allogenic growth factors or dry needling if needed, to achieve significant pain reduction and functional improvement in chronic plantar fasciitis cases. PRP is prioritized based on Herber et al.’s level I systematic review and meta-analysis, which demonstrated substantial pain reduction (SMD: 3.42, P < .00001) compared to ESWT, corticosteroids, and placebo, alongside improved foot function (AOFAS scores, P = .0009) [31]. Its ability to promote tissue regeneration through growth factors makes it an ideal choice for recalcitrant cases. Allogenic growth factors, supported by Kandil et al.’s level I randomized controlled trial showing an 87% pain reduction and 62% functional improvement (P < .001), are recommended as a strong alternative, particularly for patients seeking high satisfaction rates (92%) [41]. Dry needling, backed by Llurda-Almuzara et al.’s systematic review and meta-analysis showing short-term pain reduction (MD -1.70, P < .05), is advised if PRP or growth factors are unavailable or ineffective [27]. Other options, such as amniotic membrane (Nakagawa et al., P = .02), calcium phosphate (Matthew et al., P < .05), PDRN (Lee et al., P = .523), dextrose prolotherapy (Raissi et al.), and perforating fat injections (Gusenoff et al., P < .05), are effective but secondary due to less robust or shorter-term outcomes [29,33,32,39,40]. Corticosteroids (Seth et al., Level 2) and botulinum toxin A (Elizondo-Rodríguez et al., Level I) are not prioritized due to risks and lack of superiority, respectively [35,37]. This recommendation leverages PRP’s strong evidence base to ensure effective pain relief and tissue repair within 20-52 weeks. If symptoms persist, escalation to Phase 4 is necessary.

Phase 4: Last-Resort Surgical Therapies

Description: This group encompasses surgical interventions reserved for chronic, recalcitrant plantar fasciitis (beyond 52 weeks) that remains unresponsive to all prior phases discussed. These procedures are invasive, carry higher risks, and aim to permanently alter the plantar fascia or surrounding structures to achieve pain relief and functional restoration.

The rationale for categorization: Treatments such as transcatheter arterial embolization, cryosurgery, ultrasound-guided intra-arterial embolization, proximal medial gastrocnemius recession, plantar fasciotomy, calcaneal spur resection, radiofrequency microtomy, radiofrequency coblation, and endoscopic plantar fascia release (EPFR) are categorized as last resort due to their invasiveness, higher complication rates, and requirement for surgical expertise. These interventions are reserved for severe cases after Phase 1, 2, and 3 treatment options have failed.

Transcatheter arterial embolization: Transcatheter arterial embolization is a minimally invasive interventional procedure that selectively blocks small blood vessels [42]. In this procedure, embolic agents are injected through a catheter that is guided into the arteries that supply blood to the heel. Some of which are the peroneal arteries and the posterior tibial arteries. The goal of the embolization is to reduce blood flow to the area of inflammation in the case of the plantar fascia area that is inflamed, which in turn reduces pain.

A study by Gandhi et al. evaluated transcatheter arterial embolization using imipenem/cilastatin as an embolic agent to treat chronic plantar fasciitis in 10 patients who did not respond favorably to conservative therapies. The study showed 100% technical success in all patients who participated in the research. All the patients experienced effective pain relief without pain recurrence over six months, therefore eliminating the need for any additional treatments. The study finally suggests that the use of arterial embolization could be a promising alternative to surgery for chronic plantar fasciitis pain, however, more research is needed to confirm its efficacy [42].

Cryosurgery: Cryosurgery (CS) is a procedure that involves the use of extreme cold or liquid nitrogen or argon gas to disrupt pain-transmitting nerve fibers in the plantar fascia area. This procedure disrupts the nerve conduction pathway [43]. CS in this case is performed by the creation of a small incision and the use of a cryoprobe guided by imaging to freeze the targeted tissue. This happens for one to three minutes per cycle and this is repeated two to three times.

A prospective randomized study by Catal et al. compared two surgical treatments for chronic plantar fasciitis in 48 patients who didn’t respond positively to at least six months of conservative treatments. In the study, patients were randomly assigned to either EPFR or CS. Outcomes were measured using the AOFAS Ankle-Hindfoot Scale (AOFAS-AHS) at baseline, three weeks, and three, six, and 12 months post-surgery, with patient satisfaction assessed at 12 months using the Roles-Maudsley score [44].

Furthermore, both the EPFR and CS groups showed significant improvement in AOFAS-AHS scores at 12 months, but the EPFR group had significantly better scores at three months. At 12 months, 87% of the EPFR group reported excellent or good satisfaction compared to 65% in the CS group. The study concluded that both EPFR and CS statistically improved outcomes, however, EPFR provides better results and much higher patient satisfaction at three months [44].

Ultrasound-guided intra-arterial embolization: Ultrasound-guided intra-arterial embolization is the use of ultrasound to guide a catheter to arteries to deliver embolic agents that can block the blood vessels [45]. The purpose of the block to the vessel is to reduce blood flow and in turn, reduce the inflammatory mediators. In this instance, a blood vessel that can be used is the posterior tibial artery.

A level IV study by Sasaki et al. investigated ultrasound-guided intra-arterial embolization targeting abnormal neovessels as a treatment for plantar fasciitis. The study had 66 patients who did not respond to conservative treatments. Conducted between January 2020 and February 2022, this procedure involved treatment by inserting a needle into the posterior tibial artery and then delivering temporary embolic material [45].

The Numeric Rating Scale (NRS) pain score significantly decreased, while the AOFAS score improved from 65.8 before treatment to 92.8 one year after. The benefit of this therapy persisted through an average follow-up of 30.9 months, with no major adverse events reported in the study. The study suggests that ultrasound-guided intra-arterial embolization is a very effective treatment for recalcitrant plantar fasciitis [45].

Proximal medial gastrocnemius recession: Proximal medial gastrocnemius recession is a surgical procedure that involves the lengthening of the medial head of the gastrocnemius muscle. For this procedure to occur a small incision is made in the calf to release the gastrocnemius tendon to allow ankle flexibility and in turn, cause a reduction in the strain of the plantar fascia. A Silfverskiöld test is used to evaluate the tightness of the gastrocnemius [46]. 

A randomized controlled trial by Riiser et al. with level I evidence followed patients experiencing chronic plantar fasciitis and tight calf muscles for six years. This study compared two treatments. Stretching alone versus a combination of stretching and a proximal medial gastrocnemius recession surgical procedure to release the calf muscle. The results showed that those who had the proximal medial gastrocnemius recession surgery plus stretching experienced better foot function and less pain after six years compared to those who only stretched [47].

Plantar fasciotomy: Plantar fasciotomy is a surgical procedure where the plantar fascia is partially cut to relieve the tension in the plantar fascia. It is the most common type of surgical procedure done on patients with refractory plantar fasciitis [48]. A 2-3 cm incision is made and the plantar fascia is partially cut. By doing this the mechanical stress is released and the tissue remodels.

A level IV prospective case series by Colberg et al. explored the effectiveness of a plantar fasciotomy using a microdebrider coblation wand to treat chronic plantar fasciitis in 40 patients who were tracked over one year. The average patient age was 53.4 years, with their symptoms lasting about 20 months before treatment. The study found that the pain decreased significantly from an average NRS score of 4.7 before treatment to 2 or less at three and six months, and 0.7 at one year. Function improved significantly, with Foot and Ankle Disability Index (FADI), the Foot and Ankle Ability Measure for activities of daily living (FAAMA), and for sports (FAAMS) scores showing better outcomes at one year. Plantar fascia thickness was also reduced. Overall, 86% of patients had low pain (NRS ≤ 2), and 91% had good function (FAAMA ≥ 75%) [49].

Calcaneal spur resection: Calcaneal spur resection is a surgical procedure that involves the resection of the spur on the calcaneus. A calcaneal spur is a bony outgrowth on the calcaneus that can irritate the plantar fascia [50]. The presence of this irritation can lead to inflammation and pain in the plantar fascia.

A retrospective case series level IV study by Nakajima et al. examined the effectiveness of fluoroscopic and endoscopic calcaneal spur resection (CSR) without plantar fascial release (PFR) for treating plantar fasciitis with a calcaneal spur of ≥2 mm in 47 patients. Thirty-one of them were females and 16 of them were males. The average age was 56.4 years and the average BMI was 25.5. Patients were followed for an average of 2.7 years. Pain decreased significantly from a VAS score of 79.6 to 5.3, and the Japanese Society for Surgery of the Foot (JSSF) scores improved from 54.0 to 97.5. Patients resumed full weight bearing in about 4.4 days. This study concluded that endoscopic CSR without PFR effectively reduced pain in patients, improved their function, and allowed early weight-bearing. This study suggests that PFR may not be necessary for plantar fasciitis with a calcaneal spur [51].

Radiofrequency microtenotomy: Radiofrequency microtenotomy is a procedure that makes use of radiofrequency energy to create small lesions in the plantar fascia. The probe delivers thermal energy to the plantar fascia and that causes the ablation of the tissue which in turn reduces pain and restores function [52].

A level 2 systematic and meta-analysis review study by Thor et al. reviewed the effectiveness of radiofrequency microtenotomy for the treatment of plantar fasciitis by analyzing 11 relevant articles from PubMed and Cochrane Databases that were searched in March 2019. The results revealed a significant average increase of 40.9 points in AOFAS scores after the procedure, indicating improved foot function. The evidence was rated as fair which is grade B to support radiofrequency microtenotomy for plantar fasciitis. However, it is important to note that the study suggested the need for more high-quality, randomized controlled trials [52].

Radiofrequency coblation: Radiofrequency coblation is a procedure that uses low-temperature radiofrequency energy to ablate the plantar fascia. The temperature can range between 40 and -70°C. The ablation of the damaged tissue will reduce the inflammation and allow the stimulation of fibroblasts and collagen remodeling. In this procedure, microdebridement is carried out in a grid-like pattern [53].

A retrospective cohort level 3 study by Koh et al. compared two surgical treatments for recalcitrant plantar fasciitis (RPF). These treatments were radiofrequency plantar fascia coblation with and without gastrocnemius recession. One hundred twenty-eight patients with tight calf muscles were identified by a clinical test and grouped. Group A had 73 patients who received radiofrequency coblation alone, while Group B had 55 patients who underwent radiofrequency coblation plus endoscopic gastrocnemius recession. The outcomes of these studies were measured using pain scores VAS, foot function (AOFAS hindfoot score), quality of life (physical and mental components of the 36-Item Short Form Health Survey), patient satisfaction, and complications [53].

Both of the studied groups showed significant improvements in pain, function, and physical health at six and 24 months post-surgery. Furthermore, patients in Group B, with added gastrocnemius recession, had lower pain scores at six months (1.7 vs. 3.0) and 24 months (0.8 vs. 1.9) compared to patients in Group A who received only radiofrequency coblation. However, at 24 months, there were no differences in function, quality of life, or satisfaction between groups. This study suggests that combining radiofrequency coblation with gastrocnemius recession provides better pain relief than coblation alone, without increased complications, though other outcomes were similar after two years [53].

Endoscopic plantar fascia release: EPFR is a surgical procedure that involves the partial resection of the plantar fascia through the aid of an endoscope. This resection relieves the mechanical stress and therefore allows healing and tissue remodeling. EPFR is becoming a much more popular option compared to open procedures for the treatment of plantar fasciitis [54].

A systematic review level I study by Ward et al. examined the outcomes of EPFR for treating recalcitrant plantar fasciitis. Twenty-six studies with 978 feet were studied using databases like MEDLINE and EMBASE, searched in May 2020. The studies had an average follow-up of about 25.6 months. The AOFAS score, reported in 18 studies, improved from an average of 55.66 before surgery to 89.6 after surgery out of 100. There were complications noted in 88 of 994 patients (8.9%), with pain recurrence being the most common which affected 41 patients (4.2%). The study concluded that although EPFR offered good clinical and functional results for persistent plantar fasciitis there were notable complications, therefore, this approach should only be considered after conservative treatments have failed. More studies comparing open, endoscopic, and non-surgical approaches are needed to determine the best treatment for recalcitrant plantar fasciitis [55].

Recommendation for Phase 4 (weeks 52+): EPFR or plantar fasciotomy, supplemented by radiofrequency coblation with gastrocnemius recession if indicated, is recommended to achieve significant pain relief and functional restoration in recalcitrant plantar fasciitis cases. EPFR is prioritized based on Ward et al.’s level I systematic review, which reported a substantial improvement in AOFAS scores (from 55.66 to 89.6, P < .05) with an 8.9% complication rate, making it a highly effective option with relatively lower risks compared to open procedures [55]. Plantar fasciotomy, supported by Colberg et al.’s case series showing a significant pain reduction (NRS 0.7 at 1 year), is a strong alternative, particularly for patients requiring open surgery [49]. Radiofrequency coblation with gastrocnemius recession, backed by Koh et al.’s study showing superior pain relief (VAS 0.8 at 24 months, P < .05), is recommended for patients with tight calf muscles, as it enhances outcomes when combined with coblation [53]. Transcatheter and ultrasound-guided intra-arterial embolization (Gandhi et al. and Sasaki et al.) are effective (100% pain relief and NRS decrease, P < .05) but are reserved for non-surgical candidates due to limited long-term data [42,45]. Calcaneal spur resection (Nakajima et al., VAS 5.3, P < .05) and radiofrequency microtenotomy (Thor et al. level 2 systematic and meta-analysis study, AOFAS increase 40.9 points, P < .05) are secondary options for specific cases, while cryosurgery (Catal et al.) is not recommended due to inferior outcomes compared to EPFR [51,52,44]. This recommendation prioritizes EPFR or fasciotomy for their robust evidence and sustained outcomes, ensuring effective management of severe plantar fasciitis.

Discussion

The management of plantar fasciitis requires a structured and evidence-based approach to ensure optimal patient outcomes.

The four-phase plantar fasciitis treatment framework discussed above organizes treatments into four distinct phases: Initial Therapies, Intermediate Therapies, Specialised Therapies, and Last-Resort Surgical Therapies, based on their invasiveness, clinical applicability, and therapeutic mechanism. 

This framework establishes a comprehensive review and evidence-based treatment framework for optimizing plantar fasciitis diagnosis and management by organizing treatments according to their risk-benefit profiles, patient suitability, and evidence of efficacy. The rationale for this categorization is to provide a stepwise progression of interventions for plantar fasciitis, which starts with low-risk, non-invasive options and escalates to more invasive treatments only when necessary. This ensures a tailored approach that minimizes harm while maximizing recovery.

Each phase addresses plantar fasciitis at different stages of severity. This is essential in guiding clinicians in selecting the most appropriate evidence-based therapies to achieve pain relief and functional improvement. The treatments within each phase are described, followed by the treatments in their category and the literature available that discusses such treatments. Finally, a comprehensive recommendation for treatment progression within each phase was supported by the provided literature. The four-phase plantar fasciitis treatment framework is a practical tool to streamline clinical decision-making, ensuring treatments are applied in a structured manner to resolve symptoms before advancing to more intensive options. Only four recommendations are provided, one for each phase, with treatments grouped by phase and also justified by the referenced studies to ensure robust, evidence-based guidance.

This article reflects a single author’s review and clinical interpretation, which may present certain limitations. Future collaboration with multiple authors is recommended.

## Conclusions

This comprehensive review and evidence-based treatment framework for optimizing plantar fasciitis diagnosis and management provides a very structured, evidence-based roadmap for the diagnosis and treatment of plantar fasciitis. This is a condition affecting millions globally and causing significant morbidity due to functional limitations as a result of the heel pain. A four-phase plantar fasciitis treatment framework categorizes 30 plantar fasciitis treatments into Initial therapies, Intermediate therapies, Specialized therapies, and Last-Resort Surgical Therapies. Initial therapies, such as home stretching and RICE, are highly effective for acute cases, supported by Level I evidence demonstrating significant pain reduction and improved function. Intermediate therapies like photobiomodulation and extracorporeal shock wave therapy address persistent symptoms, while specialized therapies, particularly platelet-rich plasma, provide robust options for chronic cases, backed by Level I studies showing superior pain relief and tissue repair. Last resort surgical interventions, such as endoscopic plantar fascia release, are reserved for recalcitrant cases, with strong evidence supporting their efficacy but notable risks.

By integrating high-quality evidence from pathophysiology, the majority of which is from 2020 to 2025, this framework empowers healthcare providers such as podiatric surgeons (foot and ankle surgeons), primary care physicians, orthopedic surgeons, physical therapists, and other medical practitioners to optimize patient outcomes, reduce recurrence, and enhance quality of life.
